# Oxalic Acid Production in *Clarireedia jacksonii* Is Dictated by pH, Host Tissue, and Xylan

**DOI:** 10.3389/fmicb.2020.01732

**Published:** 2020-08-04

**Authors:** Ronald V. Townsend, Renee A. Rioux, Mehdi Kabbage, Cameron Stephens, James P. Kerns, Paul Koch

**Affiliations:** ^1^ Department of Plant Pathology, University of Wisconsin–Madison, Madison, WI, United States; ^2^ Department of Entomology and Plant Pathology, North Carolina State University, Raleigh, NC, United States

**Keywords:** *Clarireedia jacksonii*, *Sclerotinia homoeocarpa*, oxalic acid, creeping bentgrass, xylan

## Abstract

Dollar spot is caused by the fungus *Clarireedia jacksonii* and is the most common disease of golf course turfgrass in temperate climates. Oxalic acid (OA) is an important pathogenicity factor in other fungal plant pathogens, such as the dicot pathogen *Sclerotinia sclerotiorum*, but its role in *C. jacksonii* pathogenicity on monocot hosts remains unclear. Herein, we assess fungal growth, OA concentration, and pH change in potato dextrose broth (PDB) following incubation of *C. jacksonii*. In addition, OA production by *C. jacksonii* and *S. sclerotiorum* was compared in PDB amended with creeping bentgrass or common plant cell wall components (cellulose, lignin, pectin, or xylan). Our results show that OA production is highly dependent on the environmental pH, with twice as much OA produced at pH 7 than pH 4 and a corresponding decrease in PDB pH from 7 to 5 following 96 h of *C. jacksonii* incubation. In contrast, no OA was produced or changes in pH observed when *C. jacksonii* was incubated in PDB at a pH of 4. Interestingly, *C. jacksonii* increased OA production in response to PDB amended with creeping bentgrass tissue and the cell wall component xylan, a major component of grass cell walls. *S. sclerotiorum* produced large amounts of OA relative to *C. jacksonii* regardless of treatment, and no treatment increased OA production by this fungus, though pectin suppressed *S. sclerotiorum*’s OA production. These results suggest that OA production by *C. jacksonii* is reliant on host specific components within the infection court, as well as the ambient pH of the foliar environment during its pathogenic development.

## Introduction

Dollar spot on cool-season turfgrass is caused by the fungus *Clarireedia jacksonii* (formerly *Sclerotinia homoeocarpa*) and is the most economically important disease of golf course turfgrass in temperate climates (Vargas, 1994; Salgado-Salazar et al., 2018). The pathogen causes sunken, straw-colored patches roughly 5 cm in diameter that can render playing surfaces unacceptable if left untreated (Smiley et al., 2005). Though *C. jacksonii* can infect most turfgrass species, it is most severe on creeping bentgrass (*Agrostis stolonifera* L.) when temperatures are between 15 and 32°C with relative humidity greater than 85% (Walsh et al., 1999). The broad optimal temperature range and relative lack of effective cultural practices for suppressing dollar spot have led to reliance on synthetic fungicides for disease suppression (Latin, 2011). However, widespread fungicide resistance within certain fungicide classes and the financial and environmental impacts of repeated fungicide applications targeting dollar spot have led to renewed interest in the biology of the dollar spot pathogen and its interaction with creeping bentgrass (Golembiewski et al., 1995; Tomer et al., 2015).

Oxalic acid (OA) is a known pathogenicity factor for several important plant pathogens, most notably *Sclerotinia sclerotiorum* (Godoy et al., 1990; Williams et al., 2011; Liang et al., 2015; Xu et al., 2015, 2018; McCaghey et al., 2019). Dutton and Evans (1996) reviewed OA production in plant pathogenic fungi and state that the role of OA in pathogenesis “is through acidification of host tissues and sequestration of calcium from host cell walls.” Marciano et al. (1983) observed that OA production was increased in the more virulent of two *S. sclerotiorum* isolates following inoculation on sunflower. The authors also observed that the pH of the tissue infected by the more virulent *S. sclerotiorum* strain was lower (4) compared to the pH of the tissue infected with the less virulent strain (6). Favaron et al. (2004) reported that OA produced by *S. sclerotiorum* on soybean lowered the ambient tissue pH and allowed for increased activity of other pathogenesis enzymes such as endo-polygalacturonase. Further support for the pH-altering properties of OA in *S. sclerotiorum* was provided by Xu et al. (2015). They found that oxalate-minus mutants could not infect faba bean (*Vicia faba*), pea (*Pisum sativum*), green bean (*Phaseolus vulgaris*), and soybean (*Glycine max*) at a pH of 7 but could infect certain hosts when the pH was 4.2, suggesting that OA was not needed for pathogenesis when tissue pH was already low. The importance of OA in altering tissue pH to facilitate infection has also been observed in the plant pathogen *Botrytis cinerea*, where Manteau et al. (2003) found that OA was only produced in culture at a pH above 5. Not all OA pathogenicity is related to pH modification; however, OA can also modify the redox environment of the host plant to the benefit of the pathogen (Williams et al., 2011), induce programmed cell death (Kim et al., 2008), and suppress plant defenses (Kabbage et al., 2013). Overall, the majority of OA research in *S. sclerotiorum* and other pathogens suggests that OA is produced to decrease tissue pH and make conditions more favorable for pathogen infection (Xu et al., 2018).

Research on OA production by *C. jacksonii* and its role in pathogenesis on turfgrass is limited. Venu et al. (2009) reported production of OA by *C. jacksonii* on potato dextrose agar (PDA) amended with the pH indicator bromophenol blue. They used a visual rating scale to assess the PDA color change caused by an acid produced by *C. jacksonii*, and high performance liquid chromatography (HPLC) was used to confirm that the acid present in the media was mostly OA. Subsequently, Orshinsky and colleagues reported that oxalate oxidase, a plant enzyme capable of degrading OA, is induced in creeping bentgrass challenged with *C. jacksonii* (Orshinsky et al., 2012a) and that oxalate oxidases are among the most upregulated transcripts in the dollar spot resistant “Crenshaw” cultivar of creeping bentgrass (Orshinsky et al., 2012b). More recent studies confirmed the upregulation of germin-like protein genes in creeping bentgrass in response to *C. jacksonii* infection and demonstrated that *in planta* levels of OA increased in both creeping bentgrass and the model plant *Brachypodium distachyon* when inoculated with *C. jacksonii* (RR, unpublished data).

Oxalic acid is an important pathogenicity factor in *S. sclerotiorum* and other plant pathogens but its role in *C. jacksonii* infection of creeping bentgrass remains unclear. Using a combination of *in vitro* studies, we report that *C. jacksonii* produces copious amounts of OA as previously suggested; however, this study shows that OA production is responsive to the pH of the ambient environment. We show that *C. jacksonii* OA production serves to lower the pH of its surroundings, presumably to create conditions that are more suitable for fungal growth and pathogenic development. Interestingly, *C. jacksonii* also responds to the cell wall component xylan, an abundant constituent in the walls of grasses such as creeping bentgrass. Thus, *C. jacksonii* responds to both environmental and host specific cues to regulate OA production, suggesting that this molecule may be an important virulence determinant.

## Materials and Methods

### Biological Materials

Two isolates of *C. jacksonii* were selected for use in pH experiments. Isolate 29F2-1 was collected from a golf course creeping bentgrass fairway in Madison, WI, USA in 2006, and ML715 was collected from a golf course creeping bentgrass fairway in Michigan, USA in 2000. These two isolates were taken out of −80°C long-term storage and grown on PDA for 2 weeks at 22°C. Isolates were stored on PDA at −5.0°C until use for each experiment.

A single *C. jacksonii* isolate, OJN9, isolated from symptomatic creeping bentgrass at the O. J. Noer Turfgrass Research and Education Facility in Madison, WI, and *S. sclerotiorum* isolate, SS#21, obtained from the UW-Madison teaching collection, were used for plant tissue and cell wall component-amended medium experiments. These cultures were maintained as described above for isolates 2F92-1 and ML715. “Penncross” creeping bentgrass for the plant tissue amended-medium experiments was grown from seed in a growth room with a 14-h light period at 25 ± 2°C and 10-h dark period at 22 ± 2°C. Plants were bottom-watered to prevent drought stress and received fertilizer as needed.

### Impact of pH on *Clarireedia jacksonii* Radial Growth *in vitro*

Autoclaved Difco PDA (Becton, Dickinson and Company, Sparks, MD, USA) was altered to a pH of 4, 7, or 9 following cooling of the 1-L flasks until approximately 50°C. Measurement of PDB pH was done using an accumet AB 15/15+ benchtop pH meter (Fisher Scientific, Hampton, NH, USA), which was standardized before each measurement to a pH of 4, 7, and 10. The pH was adjusted by adding filtered 1M NaOH or 1M HCl in 0.38 ml increments until the desired pH was achieved. The pH-adjusted PDA was then poured into 9-cm diameter Petri plates and allowed to solidify inside a biological safety cabinet at 22°C. The experiment was conducted as a 2 × 3 factorial with the two *Clarireedia* isolates at three pH levels (4, 7, and 9) with three replications. The entire study was repeated twice over time.

Isolates were grown on non-amended PDA for 4 days in complete darkness at 22°C prior to transferring to pH-amended media. After 4 days, a 5-mm plug was taken from the outer edge of the hyphal growth and placed mycelium-side down in the center of the corresponding pH-amended PDA plates and sealed with Parafilm (Bemis Company, Neenah, WI). The plates were then incubated at a temperature of 22°C in complete darkness and radial growth measurements were taken at 24, 48, 72, and 96 h post inoculation. Radial growth was measured using a ruler and four perpendicular measurements were taken per plate at each sampling hour and averaged to provide a mean radial growth per plate for each time point.

### Impact of pH on *Clarireedia jacksonii* Biomass and OA Production *in vitro*

Autoclaved Difco PDB (Becton, Dickinson and Company, Sparks, MD, USA) was altered to a pH of 4 or 7 following cooling of the 1-L flasks until approximately 50°C. Measurement and adjustment of PDB pH were done using the aforementioned protocol. The experiment required destructive sampling and individual bottles for each sampling time point. A total of 54,125-ml glass bottles were used to assess the two isolates, plus a non-inoculated control, and were tested at two different pH levels with three replications and three sampling times of 48, 72, and 96 h post inoculation. Autoclaved 125-ml flasks were filled with 25 ml of pH-adjusted PDB and a 5-mm-diameter agar plug was taken from the outer edge of the *C. jacksonii* culture and transferred to the corresponding bottle. Each bottle was then placed into a shaker incubation chamber in a completely randomized design. The bottles were incubated at a constant temperature of 24°C in complete darkness and agitated continuously at 90 rpm in a New Brunswick Benchtop Incubator Shaker (Edison, NJ, USA) until the designated sampling hour.

At each sampling hour, the designated bottles were removed from the shaker chamber and the pH of the PDB, OA content of the PDB, and weight of the fungal mass were assessed. The PDB pH was measured using an accumet AB 15/15+ benchtop pH meter. Fungal biomass was assessed following vacuum filtration, in which individual weigh boats and filter paper were weighed prior to filtration. The contents of the bottles were then vacuum-filtered, and the filter paper was placed in the corresponding weigh boats, which were then dried at 35°C for 3 days. After drying, the weigh boat and filter paper with fungal biomass were then weighed again to provide the final biomass weight in milligrams.

Oxalic acid was quantified using a Trinity Biotech enzymatic assay kit (Jamestown, NY, USA) following the method established by Willbur et al. (2017). The use of enzyme-based techniques has been used repeatedly for accurate OA quantification and does not require the highly specialized equipment and training that HPLC requires (Kunz et al., 2006; Kim et al., 2011). Samples (5 ml) were collected from PDB and then diluted 1:1 using the kit diluent and adjusted to a pH of between 6 and 7 using 1M HCl or 1M NaOH. The adjusted samples were then purified using activated charcoal and processed according to the kit’s instructions. Briefly, tubes containing activated charcoal and samples were shaken at ambient temperature for 5 min. The remaining liquid portion of the sample was aliquoted to microcentrifuge tubes and centrifuged for 5 min at 15,000 rpm to remove remaining charcoal. Reactions were set up in a 96-well plate with 100 μl oxalate reagent A added first, followed by 5 μl of samples or standards and 10 μl of oxalate reagent B. Following addition of all reaction components, the plate was incubated at room temperature for 5 min. Included in each microplate was an OA standard curve with known OA concentrations of 0.00, 0.25, 0.50, 1.00, and 2.00 mmol/L. The absorbance of each well at 590 nm was recorded using a Bio-Rad iMark microplate absorbance reader (Hercules, CA, USA). The slope of the standard curve line on each microplate was used to convert absorbance into OA concentration.

### Oxalate Quantification in *Clarireedia jacksonii* in Response to Host Cell Wall Components

For these experiments, the fungal phytopathogen *S. sclerotiorum* was included as a reference strain because production of OA and its use as a pathogenicity factor are well-studied in this fungus. Quarter-strength Difco PDB (Becton, Dickinson and Company, Sparks, MD, USA) was prepared by adding ¼ the manufacturer’s recommended amount of dehydrated culture medium to purified water. Plant-amended media was prepared by adding 1 mg/ml of creeping bentgrass foliar tissue prior to autoclaving. Foliar tissue was collected by allowing creeping bentgrass blades to grow above pot edges, trimming with scissors, and collecting trimmings for immediate use. Cell wall component-amended media was prepared by adding 1 mg/ml of the component (Table 1) to media prior to autoclaving. Media were prepared in 250-ml flasks, each containing 100 ml of media. After autoclaving and cooling, individual flasks were inoculated with a single agar plug taken from the active margin of a 4-day-old culture of *C. jacksonii* or *S. sclerotiorum*. Inoculated flasks were incubated for 1 week at 22°C with continuous agitation at 90 rpm in a benchtop incubator shaker (New Brunswick Scientific Co., Enfield, CT, USA). After 1 week of incubation, 2 ml of culture filtrate was collected from flasks and oxalate content was assessed. Methods for OA quantification were as previously described, with the exceptions that final reaction volumes were increased by a factor of 10 (1,000 μl oxalate reagent A, 50 μl sample or standard, and 100 μl oxalate reagent B), and absorbance readings were taken in single cuvettes using a UV-Vis spectrophotometer (Beckman Coulter, Brea, CA, USA). Additionally, only a single 0.5 mmol OA standard was used. As a result, OA readings for these experiments provide relative rather than absolute quantification across treatments. Experiments with PDB, with or without creeping bentgrass tissue added, were repeated two times with a minimum of three experimental replicates each. Experiments with cell wall components were repeated three times with three replicates each. *C. jacksonii* isolate OJN9 and *S. sclerotiorum* isolate SS#21 were used for both plant tissue and cell wall component amended medium experiments.

**Table 1:** Cell wall components used for *in vitro* oxalic acid (OA) production assays.

Component	Description	Product Number	Distributor
Cellulose	Cellulose	310697	Sigma Aldrich
Lignin	Lignin, alkali	370959	Sigma Aldrich
Pectin	Pectin	416862500	Acros Organics
Xylan	Xylan from beechwood	X4252	Sigma Aldrich

### Data Collection and Statistical Analysis

Average radial growth, fungal biomass, OA concentration, and PDB pH change were assessed after 96 h of incubation, and significant differences were separated with Fisher’s least significant difference test (*p* ≤ 0.05) using the generalized linear mixed model (GLIMMIX) procedure in SAS (version 9.3, SAS Institute Inc., Cary, NC). Linear regressions were fit to calculate fungal growth at each pH level, change in PDB pH over time, and OA concentration relative to fungal biomass production. All regressions were conducted using PROC REG in SAS and regression figures produced using Plotly Chart Studio^1^. Similarly, analysis of oxalate production by *C. jacksonii* in response to creeping bentgrass foliar tissue and cell wall components was performed using the GLIMMIX procedure in SAS. Pre-planned orthogonal contrasts were used to compare between specific fixed factors and a Dunnett’s test was used to compare treatments versus the non-amended PDB controls. Box plots in Figures 2, 4, 5 were produced using Plotly Chart Studio.

## Results

### *In vitro* Growth and OA Assays

**Figure 1:**
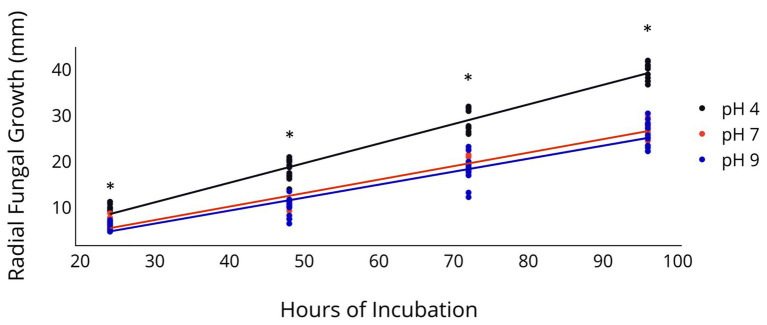
Radial Growth of *C. jacksonii* in response to medium pH. Radial growth of two *C. jacksonii* isolates on PDA at a pH of 4, 7, and 9. Colony diameter was measured 24, 48, 72, and 96 h after subculturing, and the study was repeated twice over time. No isolate or run differences were observed, so the results were combined. The black trendline indicates growth rate at pH 4 (*p* < 0.0001, *y* = 0.426*x* − 1.66, *R*^2^ = 0.955), the orange trendline growth at pH 7 (*p* < 0.0001, *y* = 0.293*x* − 1.53, *R*^2^ = 0.953), and the blue trendline growth at pH 9 (*p* < 0.0001, *y* = 0.283*x* − 1.98, *R*^2^ = 0.899). The impact of pH on radial growth was assessed at each rating date and the ^*^ indicates significance at the *p* < 0.05 level using Fisher’s least significant difference.

Radial fungal growth on PDA after 96 h was greater at a pH of 4 relative to a pH of 7 and 9 (Table 2 and Figure 1). Growth of 2F92-1 at pH 4 was 50.5% greater than at pH 7 and 46.7% greater than at pH 9, while growth of isolate ML715 at pH 4 was 39.8% greater than at pH 7 and 61.4% greater than at pH 9.

**Table 2:** Impact on of initial pH on *in vitro* radial growth on potato dextrose agar (PDA) and *in vitro* change in biomass, OA production, and decrease in pH on potato dextrose broth (PDB) of two isolates of *Clarireedia jacksonii*.

*Clarireedia* isolate	Initial pH	Radial growth (mm)^z^	Fungal biomass (mg)^z^	Oxalic acid concentration (mmol/L)^z^,^y^	Decrease in PDB pH^z^,^x^
2F92-1	4	41.13 a	15.4 a	0.0553 b	0.0156 b
	7	27.33 c	1.54 c	0.1606 a	1.533 a
	9	28.04 c	–	–	–
ML715	4	38.21 b	10.5 b	0.0624 b	0.0122 b
	7	27.33 c	2.46 c	0.1179 a	1.748 a
	9	23.67 d	–	–	–

All measurements were taken following 96 h of incubation at 22°C. Change in biomass, OA production, and PDB pH change were only assessed at a pH of 4 and 7. Each assessment was conducted in triplicate and repeated twice over time.

z Means within each column were analyzed independently. Means followed by the same letter do not significantly using Fisher’s protected least significant difference at *p* < 0.05.

y OA concentration in non-fungal controls was 0.0547 at pH 4 and 0.0519 at pH 7.

x pH in non-fungal controls increased by 0.0711 at pH of 4 and decreased by 0.0844 at pH of 7.

**Figure 2:**
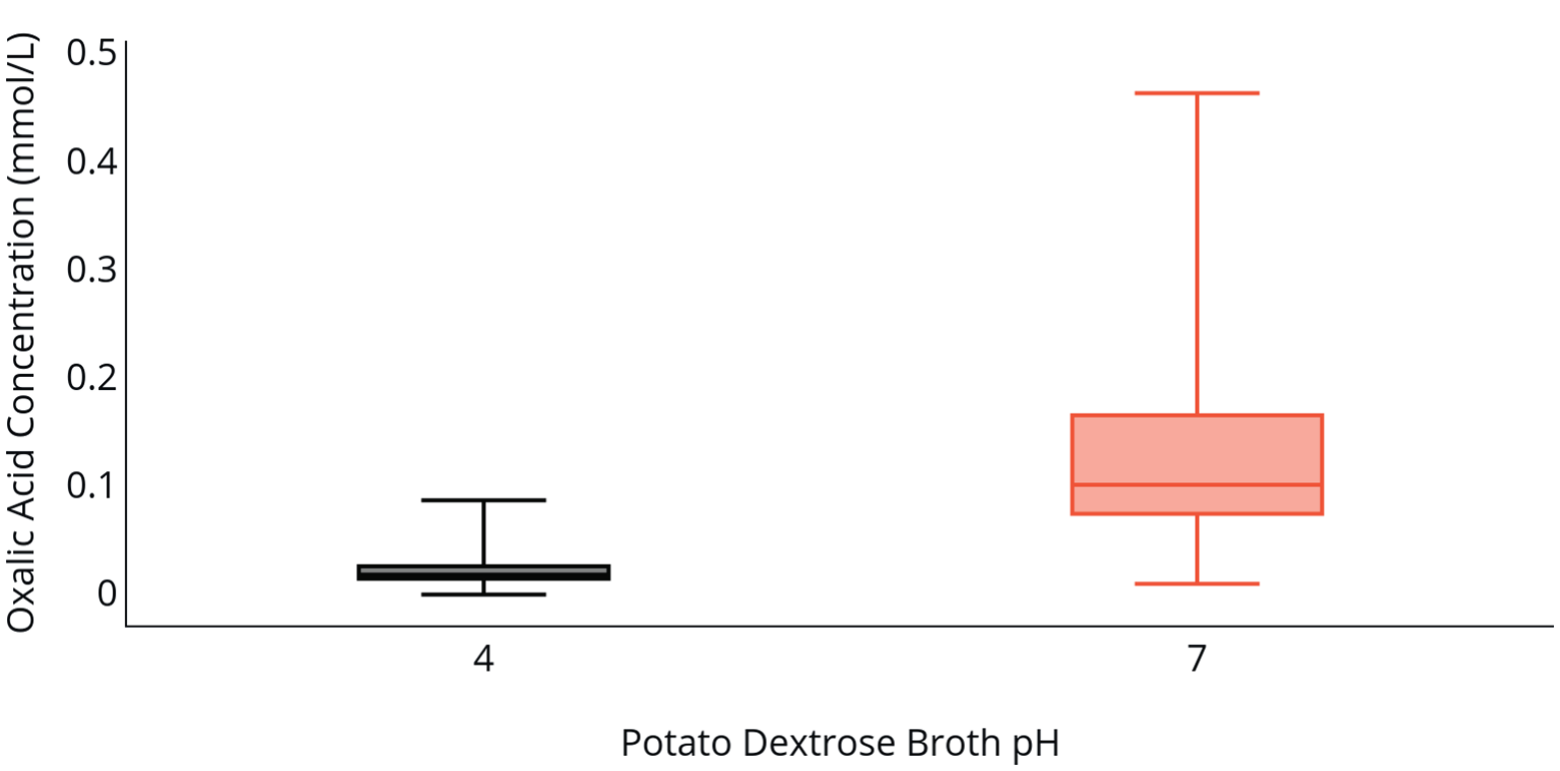
OA production by *C. jacksonii* in response to medium pH. OA concentration in PDB following incubation of two *C. jacksonii* isolates for 96 h at an initial pH of 4 (black) or an initial pH of 7 (orange). The two isolates were tested in triplicate and the study was repeated three times for a total of 36 data points. No isolate or run differences were observed, so the results were combined. The middle line within the box indicates the median OA concentration value for each pH and the top and bottom lines of the box indicate the first and third quartiles, respectively.

Fungal biomass production in PDB was greater at a pH of 4 relative to a pH of 7 following 96 h of incubation (Table 2). Isolate 2F92-1 produced 15.4 mg of biomass and isolate ML715 produced 10.5 mg of biomass at a pH of 4, but the respective isolates produced just 1.54 and 2.46 mg of biomass at a pH of 7. Concentration of OA was markedly higher at a pH of 7 relative to a pH of 4 (Table 2 and Figure 2). Oxalic acid concentration of isolate 2F92-1 was 190.4% higher at pH 7 relative to pH 4 and for isolate ML715 it was 88.9% higher at pH 7.

**Figure 3:**
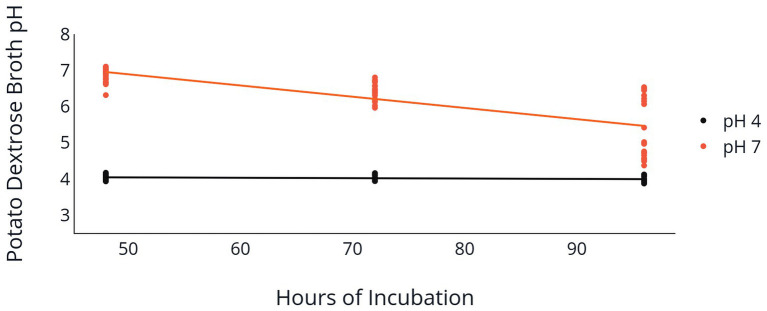
Medium pH following *C. jacksonii* inoculation. Change in PDB pH following incubation of two *C. jacksonii* isolates set at an initial pH of 4 or 7. PDB pH was measured at 48, 72, and 96 h following placement of *C. jacksonii* in the broth. No isolate differences were observed, so the results were combined. The orange trendline indicates the decline in pH in flasks initially adjusted to a pH of 7 (*p* < 0.0001, *y* = −0.031*x* + 8.45, *R*^2^ = 0.577). The black trendline indicates the decline in pH in flasks initially adjusted to a pH of 4 (*p* = 0.078, *y* = −0.001*x* + 4.09, *R*^2^ = 0.059).

Change of PDB pH over the 96 h incubation period was also influenced by the initial PDB pH. Both the 2F92-1 and ML715 isolates that began incubation at an initial pH of 7 ended with a pH near 5.5, while PDB pH of both isolates that began at pH 4 remained nearly constant over the 96 h period (Table 2 and Figure 3). Isolate 2F92-1 averaged a pH decrease of 1.533 over 96 h at an initial pH of 7 but only 0.0156 at a pH of 4, while ML715 pH decreased 1.748 over 96 h at pH 7 compared to 0.0122 at pH 4.

### Oxalate Quantification in *Clarireedia jacksonii* and *Sclerotinia sclerotiorum* in Response to Creeping Bentgrass and Host Cell Wall Components

**Figure 4:**
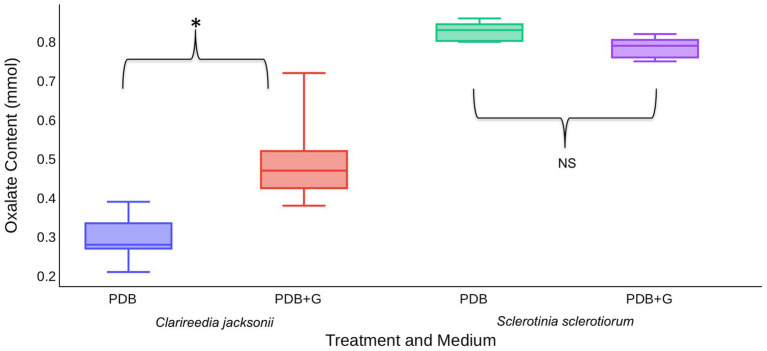
Effect of creeping bentgrass clippings on OA production by *C. jacksonii* and *Sclerotinia sclerotiorum*. OA content was quantified for culture filtrates of *C. jacksonii* isolate OJN9 and *S. sclerotiorum* isolate SS#21 grown in PDB with (PDB + G) or without (PDB) grass amendments for a 1-week period (168 h). OA production by *C. jacksonii* was significantly increased in plant tissue-amended medium (*p* < 0.001, ^*^) but similar for *S. sclerotiorum* regardless of treatment (*p* = 0.23, NS). The experiment was repeated twice over time with a minimum of three replicates per treatment in each repetition. Median and first-third quartile intervals are shown.

*In vitro* assays were used to determine if the presence of host material induces OA production by *C. jacksonii*. *S. sclerotiorum* was included in these assays to determine if induction was specific to *C. jacksonii* or a general response of OA-producing fungi. When creeping bentgrass clippings were added to ¼-strength PDB, *C. jacksonii* culture filtrates contained approximately twice as much OA than when grown in ¼-strength PDB alone (*p* < 0.01; Figure 4). *S. sclerotiorum* produced consistent amounts of OA, regardless of whether the growth medium was amended with creeping bentgrass clippings (*p* = 0.23; Figure 4).

**Figure 5:**
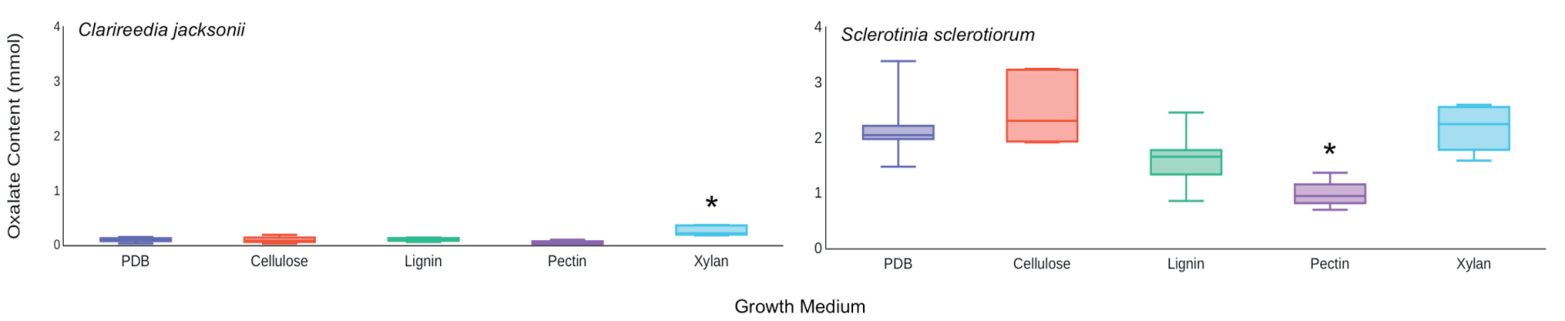
Effect of cell wall monomers on OA production by *C. jacksonii* and *S. sclerotiorum*. OA content was determined from culture filtrates collected from cultures of *C. jacksonii* isolate OJN9 **(left panel)** and *S. sclerotiorum* isolate SS#21 **(right panel)** grown for 1-week in medium with the indicated cell wall component monomer amendments. Results from the two fungi were separated due to large differences in OA production. A Dunnett’s test was used to compare OA production in cell wall component-amended media against the unamended PDB control for each fungus. Boxes with asterisks above them were significantly different from the PDB control at a significance level of *α* = 0.05. This experiment was repeated three times with three replicates per treatment in each repetition. Median and first-third quartile intervals are shown.

Based on these results, it was hypothesized that *C. jacksonii* produces OA in response to the presence of cell wall monomers. This hypothesis was tested by adding pure forms of four cell wall components (cellulose, pectin, lignin, and xylan) to PDB and quantifying the OA content of culture filtrates after 1 week of growth. Of the four cell wall components tested, xylan stimulated OA production by *C. jacksonii* (*p* = 0.05) but did not increase OA production by *S. sclerotiorum* (*p* = 0.98; Figure 5). OA production by *S. sclerotiorum* was significantly lower on pectin-amended media (*p* = 0.002) when compared to the other treatments. Pectin did not suppress OA suppression by *C. jacksonii* (*p* = 0.10; Figure 5).

## Discussion

The results presented here indicate a strong association between ambient pH and OA production in *C. jacksonii* and support earlier research from *S. sclerotiorum* and other plant pathogens that OA is used to alter ambient pH and create conditions conducive to infection. These data suggest that *C. jacksonii* prefers an acidic environment *in vitro*, which could indicate a similar preference in natural environments and during pathogenesis. Though we did not specifically test OA production and pH change in an alkaline environment, results from a neutral pH indicate that promoting a neutral and, presumably, an alkaline environment on the leaf surface could be a strategy for limiting dollar spot disease caused by *C. jacksonii*. Our research findings contribute important advancements in understanding the relationship between pH and OA production in *C. jacksonii* and underscore the need to further study these interactions in natural systems that include the host.

Limited research has been conducted for investigating the interaction of soil pH and dollar spot severity in turfgrass. Ryan et al. (2011) investigated the impact of several different fertilizer sources with different pH effects and concluded that the acidifying ammonium sulfate applications slightly reduced dollar spot, though the impact on disease was small relative to other fertilizer sources, and other studies have shown no soil pH effect on dollar spot (Markland et al., 1969). However, none of this work focused on foliar pH and the authors are not aware of any research in turfgrass investigating the impact of leaf surface pH on dollar spot severity. Given the results presented here, additional research investigating the impact of foliar pH changes on OA production and dollar spot severity in the field is warranted to further understand this potential interaction.

Oxalic acid concentration increased dramatically when *C. jacksonii* was grown at pH 7 relative to pH 4. These results support previous work by Venu et al. (2009) that found a similar pH response when qualitatively assessing OA production by *C. jacksonii* on PDA amended with the indicator dye bromophenol blue at a pH of 4 and 6. Interestingly, we observed that both radial fungal growth on PDA and biomass production in PDB slowed when grown at pH 7 relative to pH 4. The slower growth of *C. jacksonii* at the neutral pH suggests a metabolic cost to the production of OA, which *C. jacksonii* used to acidify its environment. Alternatively, the acidic environment alone may stimulate fungal growth. Acidification is known to induce the expression and activity of fungal proteases, cell wall degrading enzymes, pectinolytic enzymes, and endopolygalacturonases (Riou et al., 1991; Favaron et al., 2004; Vylkova, 2017). Overall, fungi sense ambient pH and adapt physiologically through pH-dependent signaling; these regulatory cues have been shown to influence lifestyle transitions, pathogenicity, and development (Rollins and Dickman, 2001). Despite the relatively straightforward *in vitro* impacts of pH on OA production observed in our work, *in planta* OA production and pH change are confounding factors with more complex responses. Plants work to maintain pH homeostasis as a plant defense mechanism, which will further impact OA production by the fungus and likely alter the ability of the pathogen to infect (Manteau et al., 2003). Future OA work with *C. jacksonii* should include buffered pH media to quantify OA production without the confounding factor of changing pH within the media.

In addition to OA production in response to ambient pH, we demonstrated that creeping bentgrass clippings promote OA production by *C. jacksonii* but not *S. sclerotiorum*, which produced a large amount of OA relative to *C. jacksonii* regardless of plant tissue addition. Similarly, the addition of xylan monomers to PDB promoted OA production by *C. jacksonii* with no effect on *S. sclerotiorum*. The difference in response to these media amendments by *C. jacksonii* and *S. sclerotiorum* corresponds to their distinct host ranges – *C. jacksonii* primarily infects monocots (Walsh et al., 1999) while *S. sclerotiorum* almost exclusively infects dicot species (Boland and Hall, 1994; Bolton et al., 2006). The response to xylan is also indicative of a response by this fungus to specific host cues. Xylan is a common component of all monocot cell walls but is typically only found in secondary cell walls of dicots (Faik, 2010). It is plausible that OA production by *C. jacksonii* during host infection is induced by detection or breakdown of host cell wall components, particularly xylan. Orshinsky et al. (2012b) identified genes for xylan degradation as among those most highly upregulated by *C. jacksonii* in host tissue 96 h after inoculation. Additionally, *C. jacksonii* had the most potent xylan degrading activity of 86 phytopathogenic fungi tested in a study to assess degradation of various cell wall substrates (Moscetti et al., 2013). Xylanase activity has been identified as an important virulence factor for pathogens infecting a number of cereals, including wheat, rice, and corn (Tonukari, 2000; Rajeshwari et al., 2005; Nguyen et al., 2011; Moscetti et al., 2013). Though these findings indicate a role for xylanase and connection between xylan degradation and OA in pathogenesis of *C. jacksonii*, functional characterization of xylanase genes in this fungus is needed to test these hypotheses. Interestingly, we found that pectin suppressed OA production by *S. sclerotiorum* but not *C. jacksonii*. This could have been due to a shift away from production of OA and toward that of pectinolytic enzymes, which are also a part of the *S. sclerotiorum* pathogenicity arsenal (Riou et al., 1992). Further research is needed to understand the mechanisms behind this observation.

Though the results presented here suggest that OA plays an important role in modification of the ambient environment by *C. jacksonii*, more research is needed to extrapolate these findings from *in vitro* to *in planta*. The increased production of OA by *C. jacksonii* in response to neutral pH, host tissue, and the cell wall component xylan indicates that these factors may dictate the expression of OA during pathogenesis and influences the outcomes of *C. jacksonii*-host interactions. Future research that explores the expression and role of OA during host infection by *C. jacksonii* will be critical to identifying ways in which OA can be targeted for dollar spot management. Future research should include the generation of *C. jacksonii* OA-deficient mutants to clearly assess the requirement of this molecule in pathogen infection. Similarly, interfering with the ability of *C. jacksonii* to produce OA through RNAi-based approaches may limit its capacity to lower pH within the infection court and limit disease development. It is also of interest to study the potential to manipulate the impacts of OA through modification of environmental pH as a means to provide a novel and potentially more sustainable dollar spot control strategy. Ultimately, the present research provides improved understanding of *C. jacksonii* pathogenicity mechanisms and indicates future areas of inquiry that may lead to more effective control strategies for dollar spot that reduce reliance on chemical inputs.

## Data Availability Statement

The raw data supporting the conclusions of this article will be made available by the authors, without undue reservation.

## Author Contributions

RT and RR were involved in study design, data collection, and data analysis. MK was involved in data analysis and manuscript preparation. CS was involved in manuscript preparation. JK was involved in study design and manuscript preparation. PK was involved in study design, data analysis, and manuscript preparation. All authors contributed to the article and approved the submitted version.

### Conflict of Interest

The authors declare that the research was conducted in the absence of any commercial or financial relationships that could be construed as a potential conflict of interest.

## Footnotes

^1^https://chart-studio.plot.ly/feed/#/
